# Short-and Long-term Patient Outcomes in Hospitals Primarily Serving Patients with Colorectal Cancer from High-Poverty Areas—An Observational Cohort Study

**DOI:** 10.1245/s10434-025-18816-2

**Published:** 2025-12-06

**Authors:** Xinyan Zheng, Laura C. Pinheiro, Parisa Tehranifar, Erica Phillips, Rulla M. Tamimi, Steven Y. Chao, Maria Pisu, Chuxuan Gao, Andrew G. Rundle, Jialin Mao

**Affiliations:** 1 Department of Population Health Sciences, Weill Cornell Medicine, New York, NY USA; 2 Department of Medicine, Division of General Internal Medicine, Weill Cornell Medicine, New York, NY USA; 3 Sandra and Edward Meyer Cancer Center, Weill Cornell Medicine, Cancer Prevention and Control Program, New York, NY USA; 4 Department of Epidemiology, Columbia University Mailman School of Public Health, New York, NY USA; 5 Department of Surgery, Weill Cornell Medicine, New York, NY USA; 6 Division of General Internal Medicine & Population Science, The University of Alabama at Birmingham Heersink School of Medicine, Birmingham, AL USA; 7 O’Neal Comprehensive Cancer Center, The University of Alabama at Birmingham, Birmingham, AL USA

**Keywords:** Poverty area, Colorectal cancer, Surgical outcomes, Long-term mortality, Health disparities

## Abstract

**Background:**

Prior evidence indicate that differences in treatment settings between patients with colorectal cancer (CRC) from high-poverty areas (HPA, ≥ 20% residents living under poverty level) and low-poverty areas (LPA) might have contributed to disparities in their health outcomes. We sought to determine whether certain hospitals predominantly provided surgical care for patients with CRC from HPAs and examine associated patient outcomes.

**Patients and Methods:**

We identified patients undergoing surgery for nonmetastatic CRC diagnosed during 1/1/2009–12/31/2019 from SEER-Medicare. We defined poverty-area-serving (PAS) hospitals as hospitals with ≥ 50% patients from HPAs. We compared in-hospital adverse events, 30 day readmission, and long-term mortality between patients from HPAs and LPAs treated at PAS and non-PAS hospitals using logistic and Cox regression.

**Results:**

Our cohort included 81,992 patients with CRC (median age = 78 years, 53.8% female, 15.9% in HPAs) treated by 991 hospitals. The 180 (18.2%) PAS hospitals treated 64.2% of patients from HPAs versus 2.6% from LPAs. Compared with patients from LPAs treated at non-PAS hospitals, patients from HPAs treated at PAS hospitals had more frequent in-hospital adverse events (OR[95%CI] = 1.17[1.07–1.29]), 30-day readmission (OR[95%CI] = 1.33[1.20–1.47]), worse all-cause (HR[95%CI] = 1.16[1.10–1.22]), and cancer-specific mortality (HR[95%CI] = 1.23[1.15–1.32]).

**Conclusions:**

A group of PAS hospitals treated a significant proportion of patients with CRC from HPAs and few from LPAs and was associated with worse short- and long-term patient outcomes. These findings highlight the presence and negative impact of healthcare segregation by area-level poverty and systemic inequities faced by individuals from HPAs. Multilevel resources are needed to address quality of care and other healthcare-associated needs for individuals from disadvantaged areas.

**Supplementary Information:**

The online version contains supplementary material available at 10.1245/s10434-025-18816-2.

In the USA, colorectal cancer is the third most prevalent cancer and the third and fourth deadliest cancer for men and women, respectively.^1^ High-poverty areas (HPA) (i.e., areas with 20% or more residents living under federal poverty), particularly persistent-poverty areas (PPA) that have experienced high poverty for at least 30 years, have disproportionately high colorectal cancer mortality compared with low-poverty areas (LPA).^2^ Approximately 20% of the USA population reside in HPAs.^3^ There is an imperative need to investigate factors contributing to poor cancer outcomes among individuals who reside in HPAs.

There is a growing recognition that determinants beyond individual-level factors contribute to structural inequities perpetuating health disparities. The multilevel framework proposed by the Centers for Population Health and Health Disparities points to distal factors of social conditions (e.g., poverty) and institutional context (e.g., healthcare system) as the fundamental causes of disparate health outcomes.^4^ HPAs, particularly PPAs, often experience long-term economic underdevelopment and distress.^5^ The limited economic potential inhibits opportunities and leads to inadequate infrastructure and support services, creating structural inequities in various affairs, including healthcare.^6^ Prior research has revealed that patients with colorectal cancer residing in high-poverty, low-income areas were more likely to undergo surgery at low-volume hospitals and non-NCI-designated hospitals than those from low-poverty, high-income areas.^7,8^ Being treated at low-volume and nonaccredited hospitals has been associated with worse short- and long-term health outcomes among patients with colorectal cancer.^9,10^ These pieces of evidence indicate that differences in treatment settings between patients with colorectal cancer from HPAs and LPAs might have contributed to disparities in their health outcomes. Patients from HPAs might be predominantly treated within certain treatment settings, adversely impacting their health outcomes. Empirical investigations of inequities in treatment settings are needed to guide structural interventions to reduce cancer disparities.

In this study, we sought to test the hypothesis that certain hospitals predominantly provided surgical care for patients with colorectal cancer from HPAs. Furthermore, we evaluated the impact of treatment settings and healthcare segregation on in-hospital adverse events, 30 day readmission, and long-term mortality among patients with colorectal cancer from HPAs and LPAs.

## Patients and Methods

### Data Source

We conducted an observational cohort study using the Surveillance, Epidemiology, and End Results (SEER) cancer registry data linked to Medicare fee-for-service claims (SEER-Medicare database). Specifically, we used the 2020 linkage with an update of two additional years of data. This dataset included 20 registries, covering populations in 13 states plus Detroit metropolitan and Seattle-Puget Sound areas. SEER registries collect data on newly reported cancers, including time of diagnosis, cancer site, stage, and grade. The cancer registry data also contain individuals’ vital status at the end of follow-up and the cause of death. Medicare fee-for-service claims capture inpatient and outpatient services beneficiaries receive and validated death information. About 95% of eligible Medicare beneficiaries in the SEER were successfully linked to Medicare claims.^11^

### Study Population

We identified patients who were diagnosed with nonmetastatic colorectal cancer between 1/1/2009 and 12/31/2019 and received surgery within 6 months of diagnosis (Supplementary Fig. 1). We focused on localized and regional cancer because surgery is the primary treatment for these diseases. To have a 1 year period to assess comorbidities for each patient, we included patients who were aged 66 years or older at the time of the procedure and had continuous Medicare Fee-for-Service enrollment within 1 year before the procedure. We then limited the cohort to patients who were treated at hospitals in SEER states. This is because the total number of Medicare patients treated at hospitals outside SEER states cannot be ascertained and the proportion of patients from HPAs cannot be reliably calculated. We excluded patients who were treated at hospitals with a total volume of ≤ 5 patients during the study period (*N* = 585, 0.7%) and whose county-level poverty measure was missing (*N* < 11).

### Area-level Poverty Measure

We focused on county-level poverty in this study owing to the constraints of the available data. Patients were categorized as living in high-poverty areas (HPAs) or low-poverty areas (LPAs) based on their residential county at the time of cancer diagnosis, collected by SEER. An HPA refers to an area with at least 20% of residents living below federal poverty, consistent with the definition used by federal agencies.^12^ We further divided patients from HPAs into those living in persistent-poverty areas (PPA) and those living in current-poverty areas (CPA). A PPA refers to an area that has been an HPA for the past 30 years. In this study, we used the PPA definition created by the US Census Bureau, which was based on the 1990 and 2000 Censuses and the 2005–2009 and the 2015–2019 American Community Survey (ACS) 5 year estimates.^13^ For patients who lived in an HPA during the year of diagnosis but not a PPA, we classified them as living in a CPA based on the 1 year ACS estimates when available. When the area-level poverty measure was missing in the 1 year ACS, we used the measure from the ACS 5 year estimates.

### Definition of Poverty-Area-Serving (PAS) Hospitals

We defined a poverty-area-serving (PAS) hospital as a hospital that predominantly provided surgical care to patients with colorectal cancer from HPAs. To operationalize this, we summarized the proportion of patients from HPAs for each hospital included in the study and categorized hospitals where 50% or more of the patients were from HPAs as PAS hospitals.

### Health Outcomes

The outcomes of interest were: 1) in-hospital adverse events, 2) 30 day readmission, and 3) long-term all-cause mortality, and 4) long-term cancer-specific mortality. In-hospital adverse events were a composite of death, procedural complications (bleeding, infection, injury of adjacent organs, wound complications), and medical complications (acute myocardial infarction, cardiac arrest, stroke, pulmonary embolism, deep venous thrombosis, sepsis, shock, respiratory failure, acute kidney injury). We determined in-hospital adverse events using ICD diagnosis and procedure codes (Supplementary Table 1).^14,15^ We defined readmission as a subsequent hospitalization within 30 days following the index discharge. We retrieved the date of death from the Medicare Master Beneficiary Summary File. For all-cause mortality, patients were censored at the end of the study (31 December 2020) or when they dropped out of Medicare Fee-for-Service (e.g., enrolling in Medicare Advantage), whichever was earlier. Using the SEER cause-of-death indicator, we identified patients who died of colorectal cancer. For cancer-specific mortality, patients were censored at the end of SEER follow-up, when they dropped out of Medicare Fee-for-Service, or when they died of other causes, whichever was the earliest.

### Covariates

Baseline patient characteristics examined were demographics (age at the time of the surgery, sex, race and ethnicity, urban or rural residential location, and SEER region), cancer site (colon only, rectum only, colon and rectum), cancer stage (localized or regional), cancer grade, the presence of prior noncutaneous cancers, procedure year, time from diagnosis to procedure, procedural approach (open, laparoscopic, or robotic), the use of neoadjuvant and adjuvant chemotherapy, and comorbidities. Definitions of the covariates were detailed in Supplementary Methods.

### Statistical Analysis

We described baseline patient characteristics and compared them between those from LPAs and HPAs using the Chi-squared test for categorical variables and the Wilcoxon rank sum test for age. We summarized the proportion of patients treated at PAS hospitals by area-level poverty in the entire cohort as well as by residential location (metropolitan, urban, rural) and by race and ethnicity (non-Hispanic White, non-Hispanic Black, Hispanic). We did not include other race and ethnic groups in the subgroup analysis owing to the small sample size.

We examined the risks of in-hospital adverse events and 30 day readmission by four groups jointly defined by area-level poverty (HPAs versus LPAs) and treatment at PAS hospitals. We used logistic regression to compare the differences in these two outcomes across groups. For long-term all-cause and cancer-specific mortality, we performed a Kaplan–Meier analysis and estimated the cumulative risk of death at 5 years after the procedure. We used Cox proportional hazard regression to compare differences in mortality across the four groups. For each analysis, we used an unadjusted model, an age-adjusted model, and a multivariable-adjusted model. In the multivariable model, we adjusted for age, sex, cancer site, cancer stage, cancer grade, prior noncutaneous cancer, number of comorbidities, and year of procedure. We did not adjust for procedure approach, use of chemotherapy, and time from diagnosis to procedure because they could be potential mechanisms underlying the differences in outcomes across groups. All models included a robust sandwich estimator to account for the clustering of patients within hospitals.

We performed subgroup analyses by patients’ residential urban-rural classification (metropolitan, urban, rural) and race and ethnicity (non-Hispanic White, non-Hispanic Black, Hispanic) instead of adjusting for them in the multivariable models because the impact of treatment setting on outcomes may vary across these subgroups. We performed two sensitivity analyses: 1) repeating the primary analysis for patients who received surgery within 1 year after the diagnosis, instead of using the 6 month cutoff; 2) examining patients from PPAs and CPAs separately. Odds ratios (OR) and hazard ratios (HR) were reported with 95% confidence intervals. Statistical significance was defined as *p* < 0.05 and all tests were two-sided. All analyses were performed using SAS 9.4 (Cary, NC).

## Results

A total of 81,992 patients with nonmetastatic colorectal cancer were included in the study, with 13,017 (15.9%) residing in HPAs (Table 1). The median age of patients was 78 years (interquartile range: 73–84) and 53.8% were females. Compared with patients from LPAs, those from HPAs were younger (HPA versus LPA: median age 77 versus 79, *p* < .001), more likely to be non-Hispanic Black (15.0% versus 5.6%, *p* < .001), more likely to live in rural areas (4.5% versus 1.0%, *p* < .001), and more likely to receive open surgery (63.1% versus 56.1%, *p* < .001). Patients from HPAs also had a higher number of comorbidities than those from LPAs (4+ comorbidities: 38.1% versus 33.4%, *p* < .001).

**Table 1:** Characteristics of patients undergoing surgery for nonmetastatic colorectal cancer diagnosed during 2009–2019, by county-level poverty

	Low-poverty areas	High-poverty areas	*P*-value
	(*N* = 68975)	(*N* = 13017)	
Age (Median (IQR))	79 (73, 85)	77 (72, 83)	< .001
Sex			< .001
Male	31631 (45.9%)	6220 (47.8%)	
Female	37344 (54.1%)	6797 (52.2%)	
Race and ethnicity			< .001
Non-Hispanic White	60141 (87.2%)	10389 (79.8%)	
Non-Hispanic Black	3842 (5.6%)	1955 (15.0%)	
Hispanic	846 (1.2%)	222 (1.7%)	
Other	4146 (6.0%)	451 (3.5%)	
Location			< .001
Metropolitan	61567 (89.3%)	8717 (67.0%)	
Urban	6722 (9.7%)	3714 (28.5%)	
Rural	686 (1.0%)	586 (4.5%)	
Region			< .001
Northeast	30572 (44.3%)	2247 (17.3%)	
Midwest	6199 (9.0%)	1625 (12.5%)	
South	9614 (13.9%)	6171 (47.4%)	
West	22590 (32.8%)	2974 (22.8%)	
Cancer site			0.001
Colon	58021 (84.1%)	10788 (82.9%)	
Rectum	10472 (15.2%)	2126 (16.3%)	
Colon + Rectum	482 (0.7%)	103 (0.8%)	
Cancer stage			
Localized	32954 (47.8%)	6105 (46.9%)	0.07
Regional	36021 (52.2%)	6912 (53.1%)	
Cancer grade			< .001
I	6187 (9.0%)	1087 (8.4%)	
II	49095 (71.2%)	9639 (74.0%)	
III	11816 (17.1%)	1957 (15.0%)	
IV	1877 (2.7%)	334 (2.6%)	
Prior noncutaneous cancer	12514 (18.1%)	2024 (15.5%)	< .001
Procedure year			< .001
2009–2012	28818 (41.8%)	5670 (43.6%)	
2013–2016	23870 (34.6%)	4916 (37.8%)	
2017–2020	16287 (23.6%)	2431 (18.7%)	
Diagnosis to procedure			0.16
≤ 1 month	43328 (62.8%)	8263 (63.5%)	
> 1 month	25647 (37.2%)	4754 (36.5%)	
Procedure approach			< .001
Open	38686 (56.1%)	8214 (63.1%)	
Laparoscopic	25375 (36.8%)	4084 (31.4%)	
Robotic	4914 (7.1%)	719 (5.5%)	
Neoadjuvant chemotherapy	1918 (2.8%)	442 (3.4%)	< .001
Adjuvant chemotherapy	13666 (19.8%)	2790 (21.4%)	< .001
Number of comorbidities			< .001
0	4429 (6.4%)	600 (4.6%)	
1	11911 (17.3%)	2034 (15.6%)	
2	15328 (22.2%)	2723 (20.9%)	
3	14285 (20.7%)	2701 (20.7%)	
4+	23022 (33.4%)	4959 (38.1%)	
Coronary artery diseases	27113 (39.3%)	5593 (43.0%)	< .001
Hypertension	59189 (85.8%)	11507 (88.4%)	< .001
Congestive heart failure	16064 (23.3%)	3346 (25.7%)	< .001
Diabetes	26286 (38.1%)	5568 (42.8%)	< .001
Chronic pulmonary disease	22400 (32.5%)	4705 (36.1%)	< .001
Morbid obesity	11924 (17.3%)	2523 (19.4%)	< .001
Chronic kidney disease	13467 (19.5%)	2623 (20.2%)	0.1
Peripheral vascular disease	21352 (31.0%)	4216 (32.4%)	0.001

### Existence of PAS Hospitals and Inequities in Treatment Settings

**Fig. 1:**
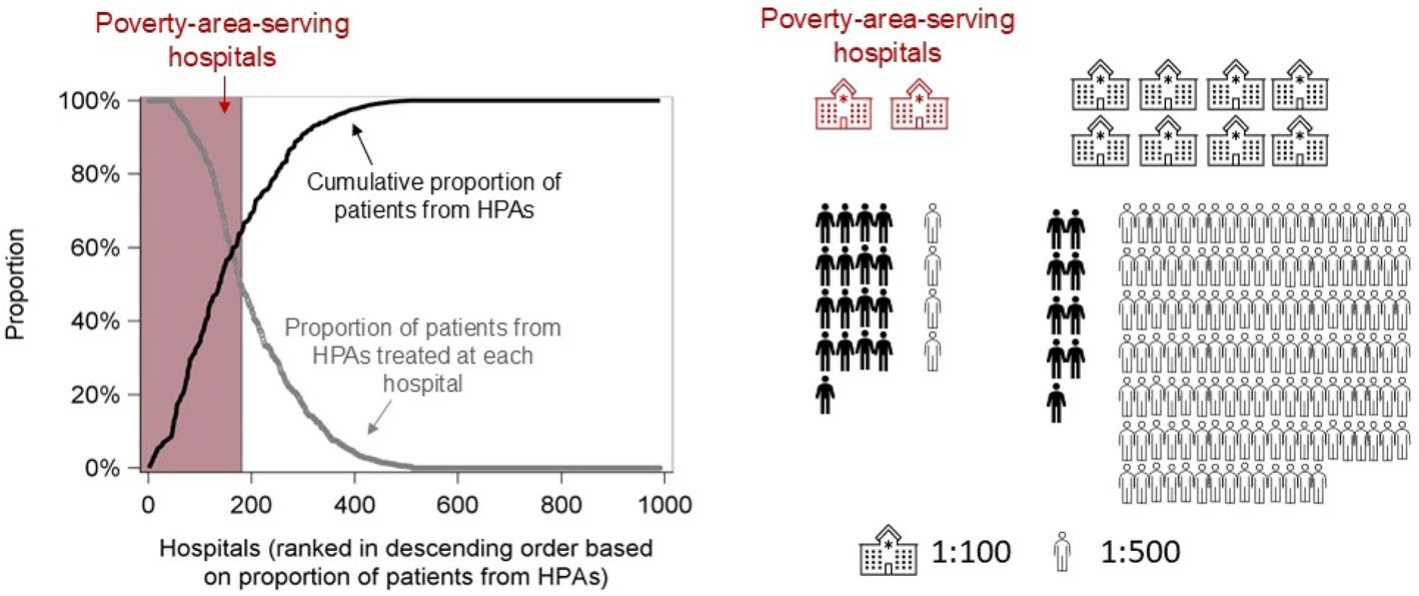
The number of hospitals predominantly treating patients with colorectal cancer from high-poverty areas (HPAs) and the proportion of patients undergoing surgery at these poverty-area-serving hospitals. Left panel:Pink shade: poverty-area-serving hospitals that predominantly treated patients with colorectal cancer from HPAs such that ≥ 50% of their patients were from HPAs;Gray dots: proportion of patients from HPAs treated at each hospital;Black line: cumulative proportion of patients from HPAs.Right panel:Hospitals are depicted in a 1:100 ratio, with red-outlined representing poverty-area-serving hospitals. Patients are depicted in a 1:500 ratio, with filled figures representing individuals from HPAs

**Fig. 2:**
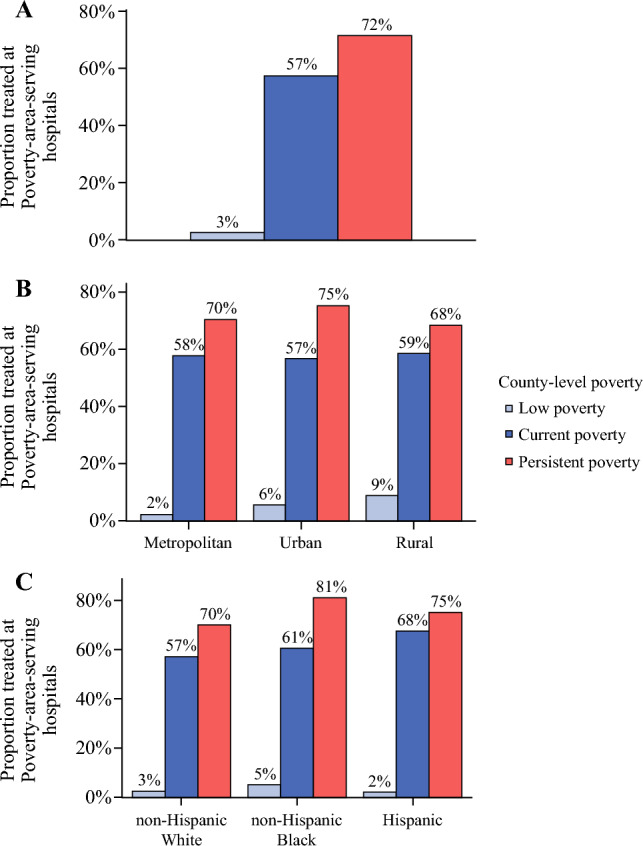
Proportions of patients with colorectal cancer from low, current, and persistent poverty areas undergoing surgery at poverty-area-serving hospitals in the entire cohort (**A**) and stratified by location (**B**) and racial and ethnic group (**C**)

The 81,992 patients were treated in 991 hospitals. Of these, 180 (18.2%) hospitals predominantly treated patients from HPAs, with 50% or more of their patients residing in HPAs (Fig. 1). In total, these PAS hospitals treated 64.2% of patients from HPAs (*N* = 8351) as opposed to 2.6% of patients from LPAs (*N* = 1821). Specifically, 71.6% of patients from PPAs and 57.5% from CPAs were treated at PAS hospitals. This pattern existed across residential locations (metropolitan, urban, and rural) and racial and ethnic groups (non-Hispanic White, non-Hispanic Black, and Hispanic) (Fig. 2).

### Health Outcomes of Patients with Colorectal Cancer from HPAs Treated at PAS Hospitals

When examining patient outcomes by area-level poverty and treatment setting, patients from HPAs treated at PAS hospitals had the worst short and long-term outcomes (Table 2). The incidence of in-hospital adverse events and 30 day readmission was 42.2 and 20.3% among patients from HPAs treated at PAS hospitals and 37.2–38.4% and 16.0–18.3% among the other three groups. The estimated 5 year all-cause and cancer-specific mortality was 46.3 and 25.8% among patients from HPAs treated at PAS hospitals and 41.0–43.1% and 22.6–23.7% among the other three groups (Supplementary Fig. 2).

**Table 2:** Association between area-level poverty, treatment at poverty-area-serving hospitals, and short- and long-term outcomes among patients undergoing surgery for nonmetastatic colorectal cancer

	LPA Non-PAS hospitals	LPA PAS hospitals	HPA Non-PAS hospitals	HPA PAS hospitals
*In-hospital adverse events*				
Events (%)	25693 (38.3%)	678 (37.2%)	1794 (38.4%)	3521 (42.2%)
Unadjusted OR (95% CI)	Reference	0.96(0.83–1.11)	1.01(0.90–1.13)	1.18(1.07–1.29)
Age-adjusted OR (95% CI)	Reference	1.02(0.88–1.17)	1.07(0.95–1.19)	1.24(1.13–1.37)
Fully adjusted OR (95% CI)	Reference	1.00(0.86–1.16)	1.01(0.89–1.15)	1.17(1.07–1.29)
*30-day readmission*				
Events (%)	10375 (16.0%)	321 (18.3%)	816 (18.2%)	1612 (20.3%)
Unadjusted OR (95% CI)	Reference	1.18(1.01–1.37)	1.17(1.07–1.27)	1.34(1.21–1.48)
Age-adjusted OR (95% CI)	Reference	1.21(1.04–1.41)	1.20(1.10–1.31)	1.38(1.25–1.52)
Fully adjusted OR (95% CI)	Reference	1.22(1.05–1.41)	1.14(1.04–1.25)	1.33(1.20–1.47)
*All-cause mortality*				
Estimated 5-year risk (95% CI)	43.1% (42.5–43.6%)	41.0% (37.4–44.8%)	42.4% (40.3–44.6%)	46.3% (44.7–48.0%)
Unadjusted HR (95% CI)	Reference	0.95(0.88–1.03)	0.97(0.91–1.03)	1.09(1.04–1.14)
Age-adjusted HR (95% CI)	Reference	1.05(0.97–1.14)	1.05(0.98–1.13)	1.20(1.14–1.26)
Fully adjusted HR (95% CI)	Reference	1.04(0.95–1.14)	1.00(0.92–1.09)	1.16(1.10–1.22)
*Cancer-specific mortality*				
Estimated 5-year risk (95% CI)	22.6% (22.0–23.2%)	23.1% (19.8–26.9%)	23.7% (21.7–25.9%)	25.8% (24.3–27.5%)
Unadjusted HR (95% CI)	Reference	1.03(0.93–1.13)	1.02(0.93–1.11)	1.16(1.09–1.25)
Age-adjusted HR (95% CI)	Reference	1.11(1.00–1.23)	1.09(1.00–1.20)	1.25(1.17–1.33)
Fully adjusted HR (95% CI)	Reference	1.11(0.99–1.25)	1.05(0.95–1.17)	1.23(1.15–1.32)

Multivariable logistic regression and Cox regression models adjusted for patient age, sex, cancer type, stage, and grade, prior noncutaneous cancer, procedure year, and number of comorbidities

LPA Low-poverty area, HPA High-poverty area, PAS Poverty-area-serving

**Fig. 3:**
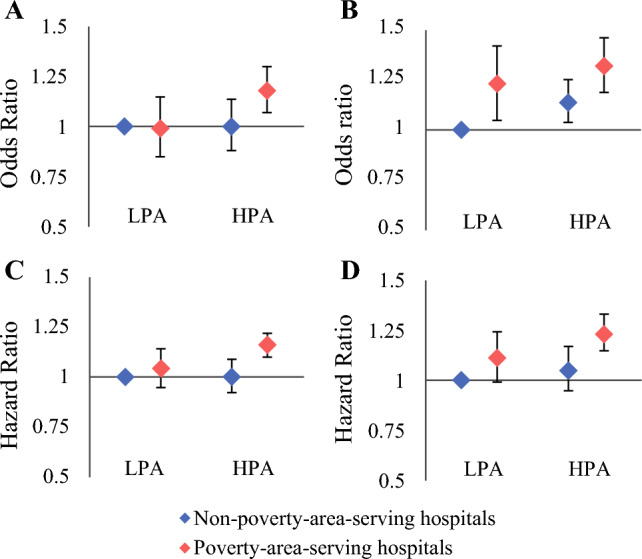
Multivariable adjusted association between area-level poverty, treatment at poverty-area-serving hospitals, and in-hospital adverse events (**A**), 30 day readmission (**B**), long-term all-cause mortality (**C**), and cancer-specific mortality (**D**) among patients undergoing surgery for non-metastatic colorectal cancer

When adjusting for demographics, cancer characteristics, procedure year, and the number of comorbidities, patients from HPAs treated at PAS hospitals had 17% higher odds of in-hospital adverse events (OR 1.17, 95% CI 1.07–1.29), 33% higher odds of being readmitted within 30 days (OR 1.33, 95% CI 1.20–1.47), 16% higher all-cause mortality (HR 1.16, 95% CI 1.10–1.22), and 23% higher cancer-specific mortality (HR 1.23, 95% CI 1.15–1.32), compared with patients from LPAs treated at non-PAS hospitals (Fig. 3). Sensitivity analysis analyzing patients who received surgery within 1 year after the diagnosis found similar results (Supplementary Table 2). We also observed similar results between patients from PPAs and those from CPAs (Supplementary Table 3).

### Subgroup Analyses by Residential Urban-Rural Classification and Racial and Ethnic Groups

In the subgroup analyses of patients with colorectal cancer residing in metropolitan and urban areas, the results were similar to those from the main analysis (Supplementary Table 4). Patients from HPAs treated at PAS hospitals had worse outcomes than those from LPAs treated at non-PAS hospitals. Among patients with colorectal cancer residing in urban areas, patients from HPAs treated at non-PAS hospitals also had worse all-cause (HR 1.08, 95% CI 0.99–1.18) and cancer-specific mortality (HR 1.10, 95% CI 0.94–1.28) than those from LPAs treated at non-PAS hospitals with a smaller magnitude, which did not meet statistical significance. The subgroup of rural residents was small and the analysis did not show any statistically significant association between area-level poverty, treatment at PAS hospitals, and health outcomes. Findings from subgroup analyses by race and ethnicity were also consistent with the main findings (Supplementary Table 5). Among Hispanic patients with colorectal cancer, we observed a higher magnitude in the increase of mortality among patients from HPAs treated at PAS hospitals compared with those from LPAs treated at non-PAS hospitals (All-cause mortality: HR 1.45, 95% CI 1.18–1.79; cancer-specific mortality: HR 1.59, 95% CI 1.17–2.17).

## Discussion

In this study, we found that a group of PAS hospitals treated a significant proportion of patients with colorectal cancer residing in HPAs and few patients from LPAs. Patients with colorectal cancer residing in HPAs who were treated at PAS hospitals had worse in-hospital outcomes, 30 day readmission rates, and long-term mortality compared with those residing in LPAs treated at non-PAS hospitals. Our findings were consistent across subgroups by residential locations (metropolitan, urban) and by racial and ethnic groups (non-Hispanic White, non-Hispanic Black, and Hispanic).

Our finding that certain PAS hospitals treated a significant proportion of patients with colorectal cancer residing in HPAs and few in LPAs indicated the presence of healthcare segregation by area-level poverty in colorectal cancer care. To our knowledge, this is the first study describing the hospital-level segregation experienced by older patients with colorectal cancer living in HPAs. Furthermore, we found that patients with colorectal cancer living in HPAs who were treated at PAS hospitals—those experiencing healthcare segregation—had worse short- and long-term health outcomes. The poorer short-term outcomes experienced by these patients may be related to the worse quality of surgical care at these hospitals. Prior research showed that patients from HPAs were more likely to receive colorectal cancer surgery at low-volume, non-NCI-designated hospitals than those from LPAs.^7,8^ PAS hospitals may have lower case volume, lower capacity of advanced technology, limited ICU support, and fewer surgical oncologists, contributing to the increased risks of in-hospital outcomes and 30 day readmission. Notably, we also found that patients with colorectal cancer from HPAs treated at PAS hospitals had higher long-term mortality compared with those from LPAs treated at non-PAS hospitals. These findings suggest that patients from HPAs who experienced healthcare segregation may be subject to lower quality of care beyond the surgical component, which affects longer-term outcomes. For example, appropriate adjuvant therapy, multidisciplinary care, and surveillance colonoscopy have been shown to have a positive impact on patient survival after colorectal cancer surgery.^16–18^ Our future work will evaluate whether these care components partially explain the worse long-term outcomes among those experiencing healthcare segregation and identify gaps in cancer care among underserved populations.

Our finding demonstrated inequities in treatment settings by area-level poverty and healthcare segregation existed across metropolitan, urban, and rural areas as well as non-Hispanic White, non-Hispanic Black, and Hispanic populations. Such inequities in treatment settings reflect systemic inequities, which likely encompass multiple aspects, including but not limited to the distribution of healthcare and social resources. For example, HPAs have lower accessibility to accredited cancer centers and specialists, and thus, have limited high-quality care options.^19,20^ As a result of social and economic policies, hospitals in low-income communities, many of which may be PAS hospitals, have lower access to capital for the construction and modernization of hospitals and expansion of health technology and services that could improve the quality of care.^21^ In addition, prior research also found that there is a lower availability of social services in HPAs versus LPAs in high and middle-density areas, which may limit the opportunities for individuals to overcome practical barriers, such as transportation, dependent care, and navigating the complex healthcare system.^22,23^ These and other interconnected factors create unequal opportunities in healthcare access and quality, underpinning disparities in care delivery and health outcomes among patients with colorectal cancer from HPAs and LPAs. While many barriers are manifested on the individual level (e.g., challenges in accessing and coordinating care), resources to address them often take root on the societal, community, and interpersonal levels. Future research is needed to identify multilevel strategies to disrupt the systemic inequity, improve quality of care and address other healthcare-associated needs for individuals from disadvantaged areas, and reduce health disparities.

Our study had several limitations. First, owing to the encryption of hospital identifiers in the SEER-Medicare, we cannot directly link to external data, such as American Hospital Association Annual Survey, to identify these PAS hospitals. However, we are conducting a follow-up study using ancillary datasets of SEER-Medicare to determine characteristics, patient composition, and resources that may be lacked at PAS hospitals. Second, only county-level poverty was available, which might result in misclassifications, particularly in densely populated metropolitan areas where a county comprises neighborhoods of varying poverty levels. Third, sample sizes of some subgroups (e.g., rural residents and Hispanic individuals) were limited and thus, results had more uncertainty. We also could not examine Asian or Native American subgroups owing to the small sample size. Forth, our study only included Medicare fee-for-service beneficiaries aged above 65 years. The generalizability to a younger population or individuals insured by Medicare Advantage cannot be ascertained. However, hospitals that predominantly treat Medicare patients from HPAs likely predominantly treat patients of any age group from HPAs.

## Conclusions

Our study demonstrated that a group of PAS hospitals treated a significant proportion of patients with CRC from HPAs and few from LPAs and was associated with worse short- and long-term patient outcomes. These findings suggest the presence and negative impact of healthcare segregation by area-level poverty and underscore systemic inequities faced by individuals from HPAs. There is a critical need for multilevel resources to address quality of care and other healthcare-associated needs for individuals from disadvantaged areas.

## Supplementary Information

Below is the link to the electronic supplementary material.

Supplementary file1 (DOCX 40 KB)

Supplementary file2 (DOCX 441 KB)

## Data Availability

The data supporting this study’s findings are not publicly available due to data use agreements. Researchers may request access to the data through the SEER-Medicare program by submitting a proposal. More information on the application process and requirements is available at: https://healthcaredelivery.cancer.gov/seermedicare/obtain/
