# Supplementary material for: Short-and Long-term Patient Outcomes in Hospitals Primarily Serving Patients with Colorectal Cancer from High-Poverty Areas—An Observational Cohort Study
Source: Ann Surg Oncol. 2025 Dec 6;33(4):3488–96. doi: 10.1245/s10434-025-18816-2 (PMC12982262; doi:10.1245/s10434-025-18816-2)
Supplement: Supplementary file 2 — Supplementary file2 (DOCX 441 KB) [file 10434_2025_18816_MOESM2_ESM.docx]

**Short and Long-term Patient Outcomes in Hospitals Primarily Serving Colorectal Cancer Patients from High-Poverty Areas**

**Supplementary materials**

**Supplementary Methods.** Definitions of baseline patient characteristics

**Supplementary Table 1.** Diagnosis and procedure codes used to identify in-hospital adverse events.

**Supplementary Table 2.** Association between area-level poverty, treatment at poverty-area-serving hospitals, and short- and long-term outcomes among patients undergoing surgery within 1 year of diagnosis for non-metastatic colorectal cancer.

**Supplementary Table 3.** Multivariable adjusted analysis of association between area-level poverty (low, current, persistent-poverty), treatment at poverty-area-serving hospitals, and short- and long-term outcomes among patients undergoing surgery for non-metastatic colorectal cancer.

**Supplementary Table 4.** Multivariable adjusted analysis of association between area-level poverty (high vs. low), treatment at poverty-area-serving hospitals, and short- and long-term outcomes among patients undergoing surgery for non-metastatic colorectal cancer, stratified by location.

**Supplementary Table 5.** Multivariable adjusted analysis of association between area-level poverty (high vs. low), treatment at poverty-area-serving hospitals, and short- and long-term outcomes among patients undergoing surgery for non-metastatic colorectal cancer, stratified by racial and ethnic groups.

**Supplementary Figure 1**. Flow chart demonstrating cohort selection and inclusion and exclusion criteria.

**Supplementary Figure 2**. Kaplan Meier curves of overall survival (A) and cancer-specific survival (B) for colorectal cancer patients by area-level poverty and treatment at poverty-area-serving or non-poverty-area-serving hospitals.

**Supplementary Methods**. Definitions of baseline patient characteristics

Urban-rural residential location was defined based on county-level rural-urban continuum (2013) codes linked by SEER. Counties in metro areas were defined as metropolitan areas (codes 1-3). Counties with urban population of 2,500 or more were defined as urban areas (codes 4-7). Counties completely rural or with less than 2,500 urban population were defined as rural areas (codes 8-9). For patients with more than one colorectal cancer diagnosis, cancer stage and grade were determined by the most advanced tumor. Days from diagnosis to procedure were categorized as within and beyond one month. We identified neoadjuvant chemotherapy administered from four months before the procedure or the time of diagnosis, whichever was later, to the time of procedure. We identified adjuvant chemotherapy administered within six months following the procedure. Chemotherapies were identified using ICD and CPT procedure codes and revenue center codes as in prior studies. ^1,2^ We identified the following comorbidities within one year before the procedure using ICD diagnosis codes based on Elixhauser comorbidity index: coronary artery diseases, hypertension, congestive heart failure, diabetes, chronic pulmonary disease, morbid obesity, chronic kidney disease, and peripheral vascular disease.^3^ We summarized the number of comorbidities for each patient and categorized the counts by 0, 1, 2, 3, and 4 or more.

1. Green SL, Dawe DE, Nugent Z, Cheung WY, Czaykowski PM. The use of chemotherapy in older patients with stage II and III colon cancer: Variation by age and era of diagnosis. *J Geriatr Oncol.* 2019;10(1):132-137.

2. Aquina CT, Brown ZJ, Beane JD, et al. Disparities in access to care among patients with appendiceal or colorectal cancer and peritoneal metastases: A medicare insurance-based study in the United States. *Front Oncol.* 2022;12:970237.

3. Elixhauser A, Steiner C, Harris DR, Coffey RM. Comorbidity measures for use with administrative data. *Med Care.* 1998;36(1):8-27.

| **Supplementary Table 1.** Diagnosis and procedure codes used to identify in-hospital adverse events. | | |
| --- | --- | --- |
|  | **ICD-9** | **ICD-10** |
| **Acute myocardial infarction** | 410.x | I21.x, I22.x |
| **Cardiac arrest** | 427.5, 799.1 | I46.x, R09.2 |
| **Stroke** | 997.02, 430, 431, 432.x, 433.x, 434.x, 438.x | G97.32, I97.811, I97.821, I60.x, I61.x, I62.x, I63.x, I65.x, I66.x, I69.x |
| **Deep venous thrombosis** | 453.4x | I82.4x |
| **Pulmonary embolism** | 415.1x | I26.x |
| **Sepsis** | 995.91, 995.92, 785.52, 998.02, 038.x | A02.1, A22.7, A26.7, A32.7, A40.x, A41.x, A42.7, A54.86, B37.7, T81.44x, R65.20, R65.21, T81.12x, A40.x, A41.x, A42.7 |
| **Shock** | 998.0x, 785.5x | T81.1x, R57.x, R65.21 |
| **Respiratory failure** | 518.0, 518.5x, 518.81, 518.82, 518.84, 96.7x | J98.1x, J95.82x, J96.0x, J96.2x, J95.2, J96.0x, R06.03, J80, J96.2x, 5A1935Z, 5A1945Z, 5A1955Z |
| **Acute kidney failure** | 584.x | N17.x |
| **Bleeding** | 998.11, 998.12, 596.7, 285.1, 569.81 | D78.02, D78.22, D78.32, E36.02, E89.811, E89.821, I97.42, I97.62, I97.620, I97.621, K91.61, K91.840, K91.870, L76.02, L76.22, L76.32, M96.811, M96.831, M96.841, N99.62, N99.821, N99.841, D62, K66.1 |
| **Infection** | 998.5x, 567.1, 567.2x, 567.3x, 567.8x, 567.9 | K68.11, T81.4x, K65.x, K68.12, K68.19, K68.9 |
| **Injury to abdominal and pelvic blood vessels** | 902.0, 902.1, 902.2, 902.3, 902.4, 902.51, 902.52, 902.53, 902.54, 902.55, 902.56, 902.50, 902.59, 902.81, 902.82, 902.87, 902.89, 902.9 | S35.0x, S35.1x, S35.2x, S35.3x, S35.4x, S35.51x, S35.53x, S35.50x, S35.59x, S35.8x, S35.9x |
| **Accidental laceration or puncture** | 998.2, 863.5x, 867.0, 867.1, 867.2, 867.3, 867.6-867.9, E870.0, E870.8, E870.9, 46.75, 48.71, 50.61, 51.91, 55.81, 56.82, 57.81 | D78.12, E36.12, G97.49, I97.52, K91.71, L76.12, M96.821, N99.72, S36.5x, S36.6x, S37.2x, S37.3x, S37.1x, S37.8x, S37.9x, 0DQEx, 0DQFx, 0DQHx, 0DQKx, 0DQLx, 0DQMx, 0DQNx, 0DQPx, 0FQ0x, 0FQ1x, 0FQ2x, 0FQ4x, 0TQ0x, 0TQ1x, 0TQ3x, 0TQ4x, 0TQ6x, 0TQ7x, 0TQBx |
| **Wound complication** | 998.30, 998.31, 998.32, 998.83 | T81.30x, T81.31x, T81.32x |

| **Supplementary Table 2.** Association between area-level poverty, treatment at poverty-area-serving hospitals, and short- and long-term outcomes among patients undergoing surgery within 1 year of diagnosis for non-metastatic colorectal cancer. | | | | |
| --- | --- | --- | --- | --- |
|  | **LPA**  **Non-PAS hospitals** | **LPA  PAS hospitals** | **HPA**  **Non-PAS hospitals** | **HPA  PAS hospitals** |
| **In-hospital adverse events** |  |  |  |  |
| Events (%) | 26100 (38.2%) | 692 (37.5%) | 1828 (38.4%) | 3548 (42.1%) |
| Unadjusted OR (95% CI) | Reference | 0.97(0.84–1.12) | 1.01(0.90–1.13) | 1.18(1.07–1.29) |
| Age-adjusted OR (95% CI) | Reference | 1.03(0.89–1.19) | 1.06(0.95–1.19) | 1.24(1.13–1.36) |
| Fully adjusted OR (95% CI) | Reference | 1.01(0.87–1.17) | 1.01(0.89–1.15) | 1.17(1.07–1.29) |
|  |  |  |  |  |
| **30-day readmission** |  |  |  |  |
| Events (%) | 10651 (16.1%) | 330 (18.5%) | 842 (18.4%) | 1639 (20.4%) |
| Unadjusted OR (95% CI) | Reference | 1.18(1.01–1.38) | 1.17(1.07–1.27) | 1.33(1.21–1.47) |
| Age-adjusted OR (95% CI) | Reference | 1.22(1.04–1.42) | 1.20(1.10–1.31) | 1.37(1.24–1.51) |
| Fully adjusted OR (95% CI) | Reference | 1.22(1.04–1.42) | 1.14(1.04–1.25) | 1.33(1.20–1.47) |
|  |  |  |  |  |
| **All-cause mortality** |  |  |  |  |
| Estimated 5y-risk (95% CI) | 43.1% (42.5-43.6%) | 41.0% (37.4-44.8%) | 42.5% (40.4-44.7%) | 46.3% (44.7-48.0%) |
| Unadjusted HR (95% CI) | Reference | 0.95(0.88–1.02) | 0.97(0.91–1.03) | 1.09(1.04–1.15) |
| Age-adjusted HR (95% CI) | Reference | 1.05(0.97–1.13) | 1.05(0.99–1.13) | 1.19(1.14–1.25) |
| Fully adjusted HR (95% CI) | Reference | 1.03(0.95–1.13) | 1.00(0.92–1.10) | 1.16(1.10–1.22) |
|  |  |  |  |  |
| **Cancer-specific mortality** |  |  |  |  |
| Estimated 5y-risk (95% CI) | 22.6% (22.0-23.2%) | 23.1% (19.8-26.8%) | 23.8% (21.8-26.0%) | 25.9% (24.3-27.5%) |
| Unadjusted HR (95% CI) | Reference | 1.02(0.92–1.12) | 1.02(0.93–1.11) | 1.16(1.09–1.25) |
| Age-adjusted HR (95% CI) | Reference | 1.10(0.99–1.22) | 1.10(1.00–1.20) | 1.25(1.17–1.33) |
| Fully adjusted HR (95% CI) | Reference | 1.10(0.98–1.23) | 1.06(0.95–1.18) | 1.23(1.15–1.33) |
| LPA = Low-poverty area; HPA = High-poverty area; PAS = Poverty-area-serving. | | | | |
| Multivariable logistic regression and Cox regression models adjusted for patient age, sex, cancer type, stage, and grade, prior non-cutaneous cancer, procedure year, and number of comorbidities. | | | | |
|  |  |  |  |  |

| **Supplementary Table 3.** Multivariable adjusted analysis of association between area-level poverty (low, current, persistent-poverty), treatment at poverty-area-serving hospitals, and short- and long-term outcomes among patients undergoing surgery for non-metastatic colorectal cancer. | | | | | | |
| --- | --- | --- | --- | --- | --- | --- |
|  | **LPA**  **Non-PAS hospitals** | **LPA  PAS hospitals** | **CPA**  **Non-PAS hospitals** | **CPA  PAS hospitals** | **PPA**  **Non-PAS hospitals** | **PPA**  **PAS hospitals** |
|  | **(N=67154)** | **(N=1821)** | **(N=2915)** | **(N=3936)** | **(N=1751)** | **(N=4415)** |
| **Postoperative death** | | | | | | |
| Adjusted OR (95% CI) | Reference | 1.16(0.89-1.57) | 1.13(1.01-1.61) | 1.09(1.16-1.63) | 1.22(0.68-1.46) | 1.11(1.26-1.89) |
|  |  |  |  |  |  |  |
| **Postoperative adverse events** | | | | | | |
| Adjusted OR (95% CI) | Reference | 1.00(0.86-1.16) | 1.18(1.05-1.33) | 1.27(1.12-1.43) | 0.77(0.60-0.98) | 1.10(0.98-1.23) |
|  |  |  |  |  |  |  |
| **30-day readmission** | | | | | | |
| Adjusted OR (95% CI) | Reference | 1.22(1.05-1.41) | 1.19(1.07-1.32) | 1.30(1.15-1.46) | 1.07(0.92-1.25) | 1.36(1.16-1.58) |
|  |  |  |  |  |  |  |
| **All-cause mortality** | | | | | | |
| Adjusted HR (95% CI) | Reference | 1.04(0.95-1.14) | 1.12(1.05-1.19) | 1.15(1.08-1.22) | 0.83(0.70-1.00) | 1.17(1.08-1.26) |
|  |  |  |  |  |  |  |
| **Cancer-specific mortality** | | | | | | |
| Adjusted HR (95% CI) | Reference | 1.11(0.99-1.25) | 1.16(1.06-1.28) | 1.23(1.13-1.34) | 0.90(0.74-1.11) | 1.23(1.11-1.37) |
| LPA = Low-poverty area; CPA = Current-poverty area; PPA = Persistent-poverty area; PAS = Poverty-area-serving | | | | | | |
| Logistic regression and Cox regression models adjusted for patient age, sex, cancer type, stage, and grade, prior non-cutaneous cancer, procedure year, and number of comorbidities. | | | | | | |

| **Supplementary Table 4.** Multivariable adjusted analysis of association between area-level poverty (high vs. low), treatment at poverty-area-serving hospitals, and short- and long-term outcomes among patients undergoing surgery for non-metastatic colorectal cancer, stratified by location. | | | | | |
| --- | --- | --- | --- | --- | --- |
|  | **LPA**  **Non-PAS hospitals** | **LPA  PAS hospitals** | **HPA**  **Non-PAS hospitals** | | **HPA  PAS hospitals** |
| **Metropolitan** | | | |  |  |
| N patients | 60179 | 1388 | 3175 | | 5542 |
| Postop adverse events  OR (95% CI) | Reference | 0.98(0.83-1.16) | 0.97(0.81-1.15) | | 1.16(1.04-1.30) |
| 30-day readmission OR (95% CI) | Reference | 1.30(1.13-1.51) | 1.09(0.98-1.22) | | 1.38(1.20-1.58) |
| All-cause mortality  HR (95%CI) | Reference | 1.04(0.95-1.15) | 0.97(0.86-1.08) | | 1.12(1.05-1.19) |
| Cancer-specific mortality  HR (95%CI) | Reference | 1.10(0.96-1.25) | 1.03(0.89-1.19) | | 1.19(1.10-1.30) |
|  |  |  |  | |  |
| **Urban** | | | |  |  |
| N patients | 6349 | 373 | 1291 | | 2423 |
| Postop adverse events  OR (95% CI) | Reference | 1.10(0.78-1.54) | 1.15(0.95-1.39) | | 1.23(1.05-1.45) |
| 30-day readmission OR (95% CI) | Reference | 0.86(0.59-1.26) | 1.33(1.11-1.59) | | 1.31(1.12-1.53) |
| All-cause mortality  HR (95%CI) | Reference | 0.98(0.81-1.19) | 1.08(0.99-1.18) | | 1.22(1.12-1.33) |
| Cancer-specific mortality  HR (95%CI) | Reference | 1.05(0.81-1.37) | 1.10(0.94-1.28) | | 1.31(1.16-1.48) |
|  |  |  |  | |  |
| **Rural** | | | |  |  |
| N patients | 626 | 60 | 200 | | 386 |
| Postop adverse events  OR (95% CI) | Reference | 0.78(0.41-1.48) | 0.77(0.50-1.18) | | 0.94(0.63-1.40) |
| 30-day readmission OR (95% CI) | Reference | 1.89(0.94-3.79) | 1.24(0.85-1.81) | | 1.23(0.85-1.78) |
| All-cause mortality  HR (95%CI) | Reference | 1.15(0.70-1.87) | 0.82(0.66-1.01) | | 1.07(0.88-1.29) |
| Cancer-specific mortality  HR (95%CI) | Reference | 1.72(1.03-2.88) | 0.82(0.58-1.17) | | 1.06(0.79-1.42) |
| LPA = Low-poverty area; HPA = High-poverty area; PAS = Poverty-area-serving. | | | | | |
| Logistic regression and Cox regression models adjusted for patient age, sex, cancer type, stage, and grade, prior non-cutaneous cancer, procedure year, and number of comorbidities. | | | | | |

| **Supplementary Table 5.** Multivariable adjusted analysis of association between area-level poverty (high vs. low), treatment at poverty-area-serving hospitals, and short- and long-term outcomes among patients undergoing surgery for non-metastatic colorectal cancer, stratified by racial and ethnic groups. | | | | | | | | |
| --- | --- | --- | --- | --- | --- | --- | --- | --- |
|  | **LPA**  **Non-PAS hospitals** | **LPA  PAS hospitals** | | **HPA**  **Non-PAS hospitals** | **HPA  PAS hospitals** | | | |
| **Non-Hispanic White** | | | |  |  | | | |
| N patients | 58603 | | 1538 | 3840 | | 6549 |  |  |
| Postop adverse events  OR (95% CI) | Reference | 0.96(0.82-1.12) | | 0.96(0.84-1.08) | 1.15(1.04-1.27) | | | |
| 30-day readmission OR (95% CI) | Reference | 1.20(1.01-1.42) | | 1.15(1.04-1.27) | 1.28(1.15-1.43) | | | |
| All-cause mortality  HR (95%CI) | Reference | 1.04(0.95-1.14) | | 0.99(0.91-1.09) | 1.13(1.06-1.19) | | | |
| Cancer-specific mortality  HR (95%CI) | Reference | 1.09(0.97-1.23) | | 1.04(0.93-1.17) | 1.19(1.10-1.29) | | | |
|  |  |  | |  |  | | | |
| **Non-Hispanic Black** | | | |  |  | | | |
| N patients | 3648 | | 194 | 569 | | 1386 |  |  |
| Postop adverse events  OR (95% CI) | Reference | 0.99(0.69-1.42) | | 1.21(0.96-1.52) | 1.08(0.91-1.29) | | | |
| 30-day readmission OR (95% CI) | Reference | 1.11(0.78-1.58) | | 0.95(0.74-1.21) | 1.39(1.17-1.65) | | | |
| All-cause mortality  HR (95%CI) | Reference | 0.94(0.73-1.21) | | 0.97(0.84-1.13) | 1.17(1.06-1.29) | | | |
| Cancer-specific mortality  HR (95%CI) | Reference | 1.00(0.73-1.37) | | 1.06(0.86-1.31) | 1.18(1.01-1.38) | | | |
|  |  |  | |  |  | | | |
| **Hispanic** | | | |  |  | | | |
| N patients | 828 | | 18 | 60 | | 162 |  |  |
| Postop adverse events  OR (95% CI) | Reference | 0.65(0.26-1.61) | | 1.16(0.67-2.01) | 1.07(0.74-1.57) | | | |
| 30-day readmission OR (95% CI) | Reference | 1.75(0.60-5.10) | | 1.22(0.60-2.48) | 0.92(0.58-1.45) | | | |
| All-cause mortality  HR (95%CI) | Reference | 1.28(0.55-2.99) | | 1.01(0.76-1.35) | 1.45(1.18-1.79) | | | |
| Cancer-specific mortality  HR (95%CI) | Reference | 2.28(0.77-6.71) | | 0.91(0.49-1.69) | 1.59(1.17-2.17) | | | |
| LPA = Low-poverty area; HPA = High-poverty area; PAS = Poverty-area-serving. | | | | | | | |  |
| Logistic regression and Cox regression models adjusted for patient age, sex, cancer type, stage, and grade, prior non-cutaneous cancer, procedure year, and number of comorbidities. | | | | | | | |  |

**
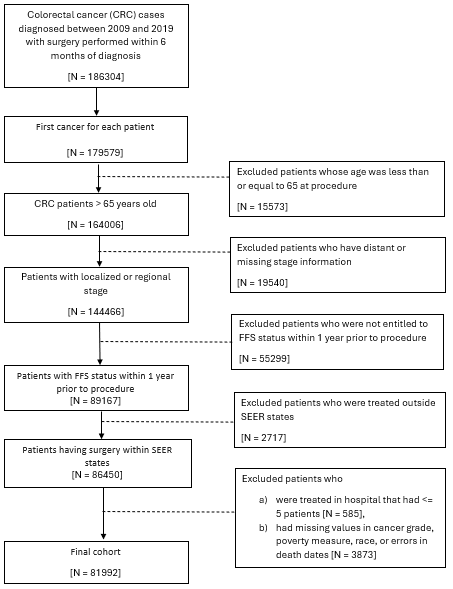
**

**Supplementary Figure 1**. Flow chart demonstrating cohort selection and inclusion and exclusion criteria.


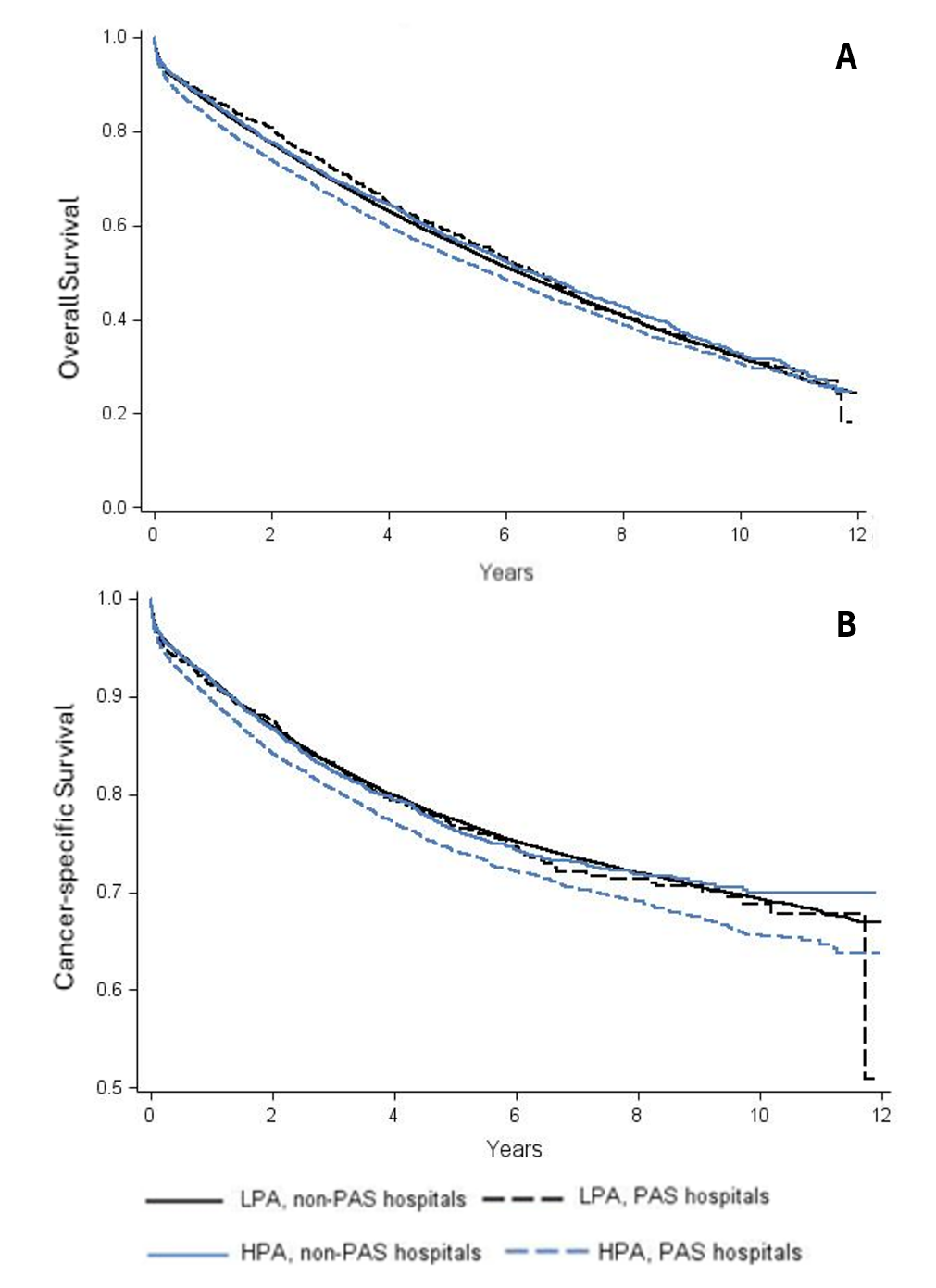


**Supplementary Figure 2**. Kaplan Meier curves of overall survival (A) and cancer-specific survival (B) for colorectal cancer patients by area-level poverty and treatment at poverty-area-serving or non-poverty-area-serving hospitals. LPA = Low-poverty area, HPA = High-poverty area, PAS = Poverty-area-serving.
